# Online group-based cognitive-behavioural therapy for adolescents and young adults after cancer treatment: A multicenter randomised controlled trial of Recapture Life-AYA

**DOI:** 10.1186/1471-2407-12-339

**Published:** 2012-08-03

**Authors:** Ursula M Sansom-Daly, Claire E Wakefield, Richard A Bryant, Phyllis Butow, Susan Sawyer, Pandora Patterson, Antoinette Anazodo, Kate Thompson, Richard J Cohn

**Affiliations:** 1 Centre for Children’s Cancer and Blood Disorders (CCC&BD), Level 1, Sydney Children’s Hospital, High Street, Randwick, NSW 2031, Australia; 2 School of Psychology, University of New South Wales, Sydney, NSW, 2052, Australia; 3 School of Psychology, Brennan MacCallum Building, The University of Sydney, Sydney, NSW, 2006, Australia; 4 Centre for Adolescent Health, Royal Children’s Hospital, 50 Flemington Rd, Parkville, VIC, 3052, Australia; 5 CanTeen, Level 11, 130 Elizabeth St, Sydney, NSW, 2000, Australia; 6 Sydney Youth Cancer Service, Medical Professorial Unit, 1st Floor South Wing Edmund Blackett Building, Prince of Wales Hospital, Barker St, Randwick, NSW, 2031, Australia; 7 OnTrac@PeterMac, Peter MacCallum Cancer Centre, Locked Bag 1, A’Beckett Street, Melbourne, VIC, 8006, Australia

**Keywords:** Adolescent and young adult, AYA, Cancer, Survivorship, Intervention study, Randomised-controlled trial, Psychological adaptation, Quality of life, Cognitive-behavioural therapy, Internet, Online

## Abstract

**Background:**

A cancer diagnosis is 2.9 times more likely to occur during the adolescent and young adult years than in younger children. This spike in incidence coincides with a life stage characterised by psychological vulnerability as young people strive to attain numerous, critical developmental milestones. The distress young people experience after cancer treatment seriously jeopardises their ability to move into well-functioning adulthood.

**Methods/Design:**

This article presents the protocol of the Recapture Life study, a phase II three-arm randomised controlled trial designed to evaluate the feasibility and efficacy of a new intervention in reducing distress and improving quality of life for adolescent and young adult cancer survivors. The novel intervention, “ReCaPTure LiFe” will be compared to a both a wait-list, and a peer-support group control. Ninety young people aged 15–25 years who have completed cancer treatment in the past 1–6 months will be recruited from hospitals around Australia. Those randomised to receive Recapture Life will participate in six, weekly, 90-minute online group sessions led by a psychologist, involving peer-discussion around cognitive-behavioural coping skills (including: behavioural activation, thought challenging, communication and assertiveness skills training, problem-solving and goal-setting). Participants randomised to the peer-support group control will receive non-directive peer support delivered in an identical manner. Participants will complete psychosocial measures at baseline, post-intervention, and 12-months post-intervention. The primary outcome will be quality of life. Secondary outcomes will include depression, anxiety, stress, family functioning, coping, and cancer-related identity.

**Discussion:**

This article reviews the empirical rationale for using group-based, online cognitive-behavioural therapy in young people after cancer treatment. The potential challenges of delivering skills-based programs in an online modality are highlighted, and the role of both peer and caregiver support in enhancing the effectiveness of this skills-based intervention is also discussed. The innovative videoconferencing delivery method Recapture Life uses has the potential to address the geographic and psychological isolation of adolescents and young adults as they move toward cancer survivorship. It is expected that teaching AYAs coping skills as they resume their normal lives after cancer may have long-term implications for their quality of life.

**Trial Registration:**

ACTRN12610000717055

## Background

### The mental health burden of cancer in adolescents and young adults

Cancer is 2.9 times more likely to be diagnosed in people aged 15–29 years than during the first 15 years of life [1,2]. Improvements in medical treatments mean that there is now a growing population of survivors in the adolescent and young adult (AYA) age range [3]. As the peak period for the onset of mental health problems coincides with the AYA period [4], a cancer diagnosis during these years can have a far-reaching impact. Well beyond treatment completion, cancer may disrupt AYAs’ ability to attain the individual and interpersonal skills that are necessary to function well as they mature [5,6]. Failing to achieve these critical milestones places young people at a significant risk for poor adaptation within their future adult roles [7].

Although most AYAs will adapt well into long-term cancer survivorship [8], as a group they experience more complex, more severe, and longer-lasting distress than do children or adults with similar diagnoses [9-11]. This distress tends to be worse after cancer treatment completion than at any other stage of their cancer trajectory [12-16]. Among long-term survivors of adolescent cancer, the prevalence of clinical disorders such as anxiety, depression, or post-traumatic stress disorder (PTSD) has been shown to be as high as 24.3%, with subclinical distress also prevalent (22.4%) [16]. Adolescent cancer survivors also report having a worse quality of life (QoL) than both survivors of childhood cancer [17], or their age-matched healthy peers [18]. Without intervention, these mental health issues pose a significant threat to survivors’ longer term psychological adjustment.

Evidence-based, age-appropriate psychosocial support for surviving AYA cancer patients is therefore strongly indicated [13]. Despite this, recent reports indicate that young people living with cancer receive little post-treatment support from the hospital environment, and find supportive care resources in community settings difficult to locate and access [19]. Consequently, AYAs report experiencing high levels of unmet needs, and subsequently, higher levels of distress [6,20-22]. This is despite the existence of evidence-based psychological therapies that could be used to assist AYAs’ positive transition to survivorship.

### Efficacy of cognitive-behavioural therapy for adolescents and young adults

Cognitive-behavioural therapy (CBT) is a ‘talking therapy’ that involves training the client to recognise, and change, maladaptive thinking styles [23]. Extensive evidence supports the efficacy of CBT in treating both anxiety [24,25] and depression [25,26] in non-cancer affected adolescents. CBT is also efficacious in preventing the development of anxiety and depression among young people who show sub-clinical distress; one meta-analysis of 130 studies showed that such programs typically achieve at least moderate sized effects [27,28].

As yet, no studies have examined the efficacy of group-based CBT in a population of AYAs with cancer. Pilot data suggests, however, that AYAs with cancer respond positively to CBT skills and are enthusiastic to incorporate these skills into their daily life [19]. Consistent with this, one recent review of interventions for AYAs with chronic illnesses (including cancer) found that multi-session, skills-based interventions can be highly effective in treating emotional or behavioural problems in this age group, yielding medium to large effect sizes [29]. Together, these data recommend CBT as a promising, yet untested, form of psychological intervention in AYAs living with cancer.

### The importance of therapy modality: group-based peer support and ‘e-therapy’ in adolescents and young adults

Peer support groups (PSGs) for cancer patients and survivors remain the most prevalent form of psychological support available [30]. However, as a form of psychological support, their effects are largely unquantified; the few that have been formally evaluated tend to show small/mixed effects among AYAs [29,31]. Despite this, PSGs may address the sense of isolation and developmental disconnection with same-aged peers created by their small, dispersed numbers while on treatment [32]. Indeed, AYAs are highly receptive to PSGs as a form of intervention [19,33,34]. The extent to which peer discussion drives positive adaptation among AYAs is therefore important to assess.

AYAs’ geographic and demographic isolation demands innovative solutions to facilitate supportive, peer-based interactions. Healthy AYAs and those living with cancer are heavy users of the internet (reporting 1–3 hours daily use), and already seek and receive many of their peer interactions online [35,36]. Computer-based ‘e-therapies’ delivered online have been hailed as a new way to overcome issues of equity in accessibility and the stigma associated with seeking help from mental health professionals for young people [37-39]. Computer-based CBT has demonstrated moderate to very large effects in treating anxiety and mood disorders among adults [40] and also appears efficacious among children and adolescents [41]. It follows, then, that CBT delivered online to AYAs recovering from cancer may be effective.

## Methods/Design

The Recapture Life-AYA study is a multi-site, phase II randomised controlled trial (RCT) to assess the feasibility and efficacy of a new, online, CBT-based intervention for AYAs after cancer treatment. AYAs aged 15–25 years at treatment completion will be recruited and randomised to one of three treatment arms: Recapture Life-AYA, a peer-support group (PSG) control, and a six week waitlist control group. The novel intervention, named **‘ReCaPTure LiFe-AYA’** (**R**esilience and **C**oping skills for young **P**eople and their families **T**o **L**ive well **F**ollowing cancer-**A**dolescent and **Y**oung **A**dult version), is delivered to groups of 3–5 AYAs by a psychologist in six, weekly online sessions, shortly after cancer treatment completion. The Recapture Life-AYA program is part of the larger Recapture Life program, which includes a Recapture Life-Parents/Carers program for caregivers of recently off-treatment paediatric cancer patients.

This study employs a 3 (treatment condition) x 3 (assessment point) factorial design. All participants will be assessed using a battery of measures at baseline (T1: recruitment), post-treatment (T2: seven weeks after T1) and 12 months following participation in Recapture Life-AYA or the PSG control (T3). These measures will include a measure of QoL, as well as measures assessing AYAs’ anxiety, depression, and family functioning.

### Study aims and hypotheses

This phase II randomised controlled trial (RCT) aims to assess:

1. The efficacy of Recapture Life-AYA in improving QoL in AYA cancer survivors. In addition to assessing QoL (the primary study outcome), secondary outcomes assessed will include depression, anxiety and family functioning.

2. The feasibility of implementing Recapture Life-AYA Australia-wide, including the recruitment procedure, response/attrition rates and cost.

We hypothesise that shortly after intervention, and at the 12 month follow up:

1. Both the PSG control and Recapture Life-AYA groups will show improved QoL and other secondary psychological outcomes compared with the waitlist control.

2. Recapture Life-AYA will improve participants’ QoL, depression, anxiety, and family functioning to a greater extent than will the PSG control group.

3. Participation in Recapture Life-AYA will prove feasible and economical.

### Participants

This study will recruit 90 AYA participants from hospitals across Australia in the first 1–6 months after treatment completion. AYAs will also have the option of inviting a support person to receive information and updates through the Recapture Life-AYA study, however as this is optional no ‘target’ sample size of support persons has been set. The AYA sample size will allow differences of a medium to large effect size (*d* = 0.65, the difference in change from T1 to T2 for any pair of groups, standardised on the pooled within-group standard deviation) on the primary outcome variable (QoL) to be detected with a power of 80% at a significance level of 0.05 (two-tailed). This effect size is clinically significant [42] and is based on other RCTs of similar interventions for AYAs with chronic illness [43-45]. It is anticipated that approximately 220 patients will need to be approached to achieve a final sample of 90 participants (assuming a response rate of 50% and attrition rate of 20%, based on previous interventions with AYA) [19,46,47].

#### Inclusion criteria

Eligible AYAs will: i) have finished cancer treatment for a primary or secondary/relapsed cancer with curative intent in the preceding 1–6 months; ii) be aged between 15–25 years (inclusive); iii) be able to read English; iv) be able to provide the name and contact details of a trusted health professional (e.g., local general practitioner, family doctor or hospital social worker); and v) be able to access the Internet in a private location (see also *Access Considerations* section below).

AYAs may also choose to invite a support person (a parent/caregiver, or spouse/partner) to take part in the study, although this is not a requirement of participation.

#### Exclusion criteria

AYAs will be excluded if during the initial intake interview they: i) have insufficient English language skills to complete the interview; ii) demonstrate extremely severe scores of depression (i.e., score > 28 on the Depression subscale of the Depression, Anxiety, Stress Scales-Short Form) [48] and/or endorse serious suicidal intent; or iii) endorse symptoms of psychosis or substance abuse.

If a participant relapses during the study period, they will be referred back to their treatment center for individual support, as their needs are likely to be difficult to manage in a group setting and can negatively affect others [49]. All excluded individuals will be referred to their treating centre for support.

#### Access considerations

Internet access and a suitable computer set-up is a requirement to participate in Recapture Life-AYA. This includes access to a computer/laptop which has a microphone and web-camera, and is located such that the participant will be able to participate in the sessions comfortably and in a private and uninterrupted capacity once per week for six weeks. AYAs without access to these resources will be able to borrow a laptop, web-camera, microphone and/or wireless internet connection device for the duration of the study (the costs of which are covered by a project grant). The equipment and internet needs of all AYA participants will be assessed at the initial telephone intake interview, and access issues managed on a case-by-case basis.

#### Participant recruitment

Potential participants aged ≥16 years will be mailed a personalised invitation letter, consent form and opt-in card by their oncologist. Parental consent to approach participants <16 years will be sought. Nominated support persons given the study information by their AYA child/partner may also choose to opt in by returning the consent form. The research assistant will contact all AYA participants who opt in to the study to request that participants sign and return a written contract agreeing to use the provided equipment solely for study purposes and to address internet access issues.

**Figure 1:**
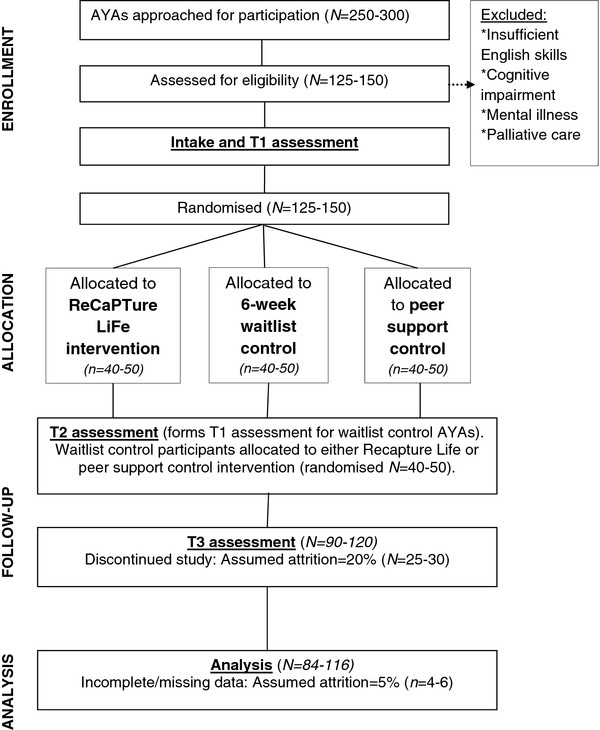
Recapture Life-AYA study flowchart.

Recruitment will occur in 3x12 week blocks, such that ~80 patients who have completed treatment in the preceding 1–6 months will be invited 4 weeks prior to Week 1 of each block. This recruitment window was chosen to maximise the benefit to participants, by delivering the coping skills intervention early in survivorship. Given the assumed response and attrition rates (see above), it is envisaged that 3 iterations (i.e. 3x12 week blocks), each attracting ~40 AYA opt-ins, will be required to achieve the target sample. This means that 3 mail outs (approaching ~80 patients at a time) will occur for the study during 2012. This design ensures the standardisation of time since treatment completion across arms. All fully consented participants will be telephoned by the study co-ordinator one week prior to Week 1 to administer the Psychosocial Adjustment to Illness Scale (PAIS; T1 assessment). Participants will also complete the first online questionnaire at this time. (See Figure 1 below, for study flowchart).

### Randomization

Participants will be randomised to one of the three study arms: a) Recapture Life-AYA, b) the PSG, or c) a waitlist condition. A flexible biased urn method of randomization, will be used, which adapts to the degree of imbalance between groups in a dynamic manner over the trial [50]. This method is superior to standard stratification in balancing groups across multiple covariates [51,52] whilst also being a suitable method when groups remain small [50,53,54]. This minimization will be based on four factors: (i) gender (male/female), (ii) severity of distress as measured by the Depression, Anxiety, Stress Scale-Short Form (DASS-21) intake interview (see below), (iii) time since treatment, classified into two groups, and (iv) degree of rural/remoteness as assessed by the Accessibility/Remoteness Index of Australia [55]. Participants will be randomised using electronic randomization used by independent personnel at the University of New South Wales.

### Interventions

#### Recapture Life-AYA intervention

Recapture Life is guided by the *Adolescent Resilience Model* (ARM) [56]. Based on extensive research in AYAs with cancer, the ARM conceptualises resilience in young people as a multifaceted process, involving the interaction of individual, family and social-level risk and protective factors to determine AYAs level of resilience, and ultimately their quality of life. Additional file 1: Table S1 identifies the key components of this model and the way in which these processes are targeted in Recapture Life. Several adaptive processes are implicated by this model including AYAs’ ability to: enact positive coping behaviours, negotiate uncertainty in illness and cancer-related distress, reappraise the experience in order to derive adaptive meaning from it, and seek and maintain support from family and friends. To our knowledge, only one other published intervention has utilised this framework in AYAs [57,58], however this music therapy intervention is delivered individually to AYAs receiving stem-cell transplants whilst on treatment. As such, this program may not offer the skill-building and peer support that we hypothesise are crucial to promote adaptation into survivorship. In addressing these component processes of resilience after cancer, our manualised program derives its core mechanisms of change from CBT techniques.

The primary goals of the Recapture Life-AYA intervention are to improve QoL, reduce distress and facilitate healthy coping in young cancer survivors. Participants randomised to Recapture Life-AYA will participate in six, weekly, 90-minute sessions facilitated by a psychologist. Each group will comprise the psychologist plus a minimum of 3 and a maximum of 5 AYAs with mixed diagnoses and genders, separated into two age groups: 15–17 and 18–25 years. Each Recapture Life-AYA module applies CBT techniques to the key domains of concern identified by our team’s previous work [59,60] (See Additional file 2: Table S2).

Sessions will be delivered through video-conferencing software on the Internet (WebEx, by Cisco). WebEx videoconferencing requires a computer with standard browser, a high-speed internet connection, and a webcam. WebEx is a secure, password-protected video-conferencing program that allows ≤6 participants to be seen on the screen simultaneously, much like Skype^TM^. Participants will also receive a reminder SMS on their cellular phone 24 hours before their session, which will also serve as their reminder to complete the emotion thermometers tool and homework compliance scale (see *Assessments* section).

Nominated support-person participants receive a workbook describing the program and one group-based 2-hour online session which includes family communication training. Support persons associated with AYAs randomly allocated to receive the Recapture Life-AYA intervention will additionally receive a weekly psycho-educational email, which includes a general overview of some of the skills learnt by the AYAs. These updates are not specific to the individual participant and include examples of (i) ways the support person can support the AYA, and (ii) suggestions for communicating about the weekly topic.

#### Peer-support group (active control)

The PSG control is delivered in an identical manner to Recapture Life-AYA (via WebEx, 3–5 participants/group, with the same facilitator, divided into 2 age groups), frequency of contact (six weekly 90-minute sessions), and the availability of peer-based group discussion. Like Recapture Life-AYA, it also involves age-appropriate supportive counseling to normalise the range of AYA experiences [61] and provides AYAs an opportunity to give and receive emotional/practical support. During each session, AYAs are encouraged to exchange information about a nominated topic (closely match those addressed in Recapture Life-AYA, e.g. ‘relationships’). The key distinction between Recapture Life-AYA and the PSG control is that the PSG does not include directive, structured teaching of specific, CBT-based coping skills. The PSG also does not include the support-person element where they receive psychoeducation and weekly emails alongside the AYA online sessions. The PSG in this trial will adhere to best practice guidelines [62] and is manualised to ensure standardization across all sessions.

### Procedures

Following recruitment, participants will complete an intake interview with the study psychologist (USD) to further screen for participant eligibility, orient participants to the study procedures, and ascertain the suitability of the participant’s internet connection. The intake interview will also serve to build rapport with the psychologist, by providing AYAs the opportunity to discuss their cancer experiences, current needs, and personal goals in participating in the study.

Participants will then be randomly allocated to one of the three study arms. The research assistant will send a hired (insured) laptop to any participants who do not have computer access two weeks before the commencement of each online group. This process will ensure equity (all participants will have access to identical equipment), minimise technical challenges (required software/hardware will be pre-installed) and will maximise security (security settings will be pre-set by the technical support person). All equipment will be returned after study participation. Participants randomised to the Recapture Life-AYA condition will also receive a written manual summarising the weekly home practice activities.

During Weeks 1–6 of each 12 week block, those allocated to Recapture Life-AYA and the PSG will participate in their allocated intervention. In Week 7, all participants will complete their second assessment (T2). For waitlist controls, this assessment will also serve as their T1 (pre-intervention) assessment. In Week 7, waitlist participants will be randomly allocated to either Recapture Life-AYA or PSG (completed during weeks 7–12). Waitlist participants will then complete a second T2 assessment, one week after their intervention.

#### Study integrity

This study is listed on the Australian New Zealand Clinical Trials Registry (ACTRN12610000717055), and has undergone rigorous multidisciplinary peer-review. It is endorsed by both the Clinical Oncology Society of Australasia’s (COSA) AYA Cancer Research Steering Committee, as well as the Scientific Advisory Committee of the Psycho-oncology Co-operative Research Group (PoCoG). In addition to having its own study consumer representatives (one male, two female, mean age: 22 years), the Recapture Life-AYA study has also been reviewed by the PC4 and PoCoG Joint Consumer Advisory Group panel, who endorsed the appropriateness and clinical importance of the research question.

Ethical approval has been obtained from the South Eastern Sydney Local Health District (SESLHD) – Northern Sector, Human Research Ethics Committee (HREC Ref: 12/008) for three sites (Centre for Children’s Cancer and Blood Disorders, Sydney Children’s Hospital; Sydney Youth Cancer Service, Prince of Wales Hospital; and the Victorian Adolescent and Young Adult Cancer Service, Peter MacCallum Cancer Centre). Ethical approval for additional sites around Australia will be submitted in July 2012. This study complies with the CONSORT guidelines [63] by using: a) standardised assessment measures; b) blind assessments; c) standardised assessor training and inter-rater reliability checks; d) manualised, replicable procedures for all conditions; e) random allocation, and f) treatment fidelity checks.

#### Treatment fidelity

Both treatment groups will be facilitated by the same person to prevent therapist confounds (e.g. therapist attributes such as age, gender and communication style, each of which could impact group retention/efficacy). Any variation or systematic biases between groups will be detected and corrected by the independent assessors during treatment fidelity checks of a random 15% of all video-recorded sessions (the validated ‘Method of Assessing Treatment Delivery’ advises a minimum of 11%) [64] All pre- and post-treatment outcome measures will be administered by the study co-ordinator, who will be blind to group allocation. In compliance with the CONSORT guidelines, the assessor will report on which condition they believe each participant was in at the beginning and end of the study.

In addition, to ascertain why this intervention may not be tolerated by all young people, exit interviews will be collected for all who leave the study prematurely, as well as 15% of completers to collect in-depth data on participants’ likes and dislikes and soliciting ideas for improvement.

#### Safety monitoring

Although research suggests that teaching AYAs CBT-based coping skills is likely to reduce their post-cancer distress [29], it is possible that some participants will experience increased short-term distress. This trial includes safety monitoring and management procedures at multiple stages of the project (See Additional file 3: Table S3, for details). Firstly, the intake interview involves careful screening for acutely suicidal/severely depressed participants at study intake using validated items sourced from the Adolescent Suicide Assessment Protocol [65].

Secondly, participants will be regularly screened for deterioration in mood during the trial when they complete the weekly emotion thermometers tool (see *Assessments,* below). Any serious deterioration in mood reported on any study assessment measures will trigger protocols involving contacting the participant to discuss their emotional state, and a meeting between the study researchers to develop a management plan (which may involve contacting their nominated health professional).

### Assessments

The following measures will be administered at all time-points (see Table 1). The study questionnaire has been reviewed by pilot testing with AYA consumers, who report that it is easy to understand and of a reasonable length (completion time <40 minutes for 135 items). Details relating to each measure are presented in Additional file 4: Table S4.

**Table 1:** Assessment schedule for the Recapture Life-AYA study

**Measure**	**Intake**	**T1**^**a**^	**During intervention**^**b**^	**T2**^**c**^	**T3**^**d**^
Psychosocial Adjustment to Illness Scale-Interview form (PAIS)	**X**	**-**	**-**	**-**	**-**
Demographic data*	**-**	**X**	**-**	**-**	**-**
Emotion thermometers tool*	**X**	**-**	**X**	**X**	**X**
Homework Compliance Scale			**X**	**-**	**-**
Impact of Cancer Scale (IOCS)			**-**	**-**	**-**
Depression, Anxiety, Stress Scales-short form (DASS-21)	**X**	**X**	**-**	**X**	**X**
Centrality of Event Scale-Short Form	**-**	**X**	**-**	**X**	**X**
Perception as “cancer survivor” item	**X**	**X**	**-**	**X**	**X**
McMaster Family Assessment Device*	**-**	**X**	**-**	**X**	**X**
KIDCOPE-Older Version	**-**	**X**	**-**	**X**	**X**
Youth Satisfaction Questionnaire*	**-**	**X**	**-**	**X**	**X**
Intervention satisfaction items*	**-**	**X**	**-**	**X**	**X**

* Measures with an asterisk also used in support-person participants at these same time-points.

^**a**^ T1 = Baseline; ^**b**^During intervention = weekly prior to intervention sessions 2–6; ^**c**^ T2 = post-intervention; ^**d**^ T3 = 12-month follow-up.

#### Demographic measures

For both AYA and support-person participants, information on age, sex, education, employment status, family structure, and the AYAs’ diagnosis and treatment regimen will be collected using standardised items adapted from the Childhood Cancer Survivor Study questionnaire items [66].

#### Psychosocial functioning

There is little consensus regarding the relative superiority of generic versus disease-specific psychosocial measures in psycho-oncology. Disease-specific measures often appeal to researchers due to the difficulty ascertaining ‘clinical’ change in populations that may only be distressed at sub-clinical levels [67,68]. However, generic psychological measures facilitate comparison to healthy norms, which is clinically and empirically advantageous [29]. As recent reviews have not shown disease-specific measures to provide a systematic advantage in detecting intervention effects [29], the Recapture Life-AYA trial uses both generic and disease-specific indicators of psychosocial functioning.

Quality of life (QoL) was selected to be the primary psychosocial outcome variable, as this enabled the measurement of specific functional issues specific to early cancer survivorship. The Impact of Cancer Scale (IOCS) [69,70] was chosen to measure QoL, and measures aspects of cancer survivorship such as: Life Challenges, Body/Health, Talking With Parents, Personal Growth, Thinking/Memory Problems, Health Literacy, Socializing and Financial Problems. Although this measure has been validated in 18–39 year old AYA cancer survivors, its readability and content relevance for the younger AYAs in the Recapture Life-AYA trial was confirmed through assessments pilot testing with consumer representatives aged 15–18 years. The Flesch-Kincaid reading level was also assessed as being at Grade 6.15.

There will be a number of secondary psychosocial functioning measures. The *Psychosocial Adjustment to Illness Scale–Interview Form (PAIS)*[71] will be administered to participants over the telephone at each time point. The PAIS assesses psychosocial adjustment of patients to cancer across seven domains including: Health Care Orientation, Vocational Environment, Domestic Environment, Sexual Relationships, Extended Family Relationships, Social Environment and Psychological Distress.

The *Depression, Anxiety and Stress Scale-Short Form (DASS–21)*[48] is included as a brief, generic measure of other relevant symptoms of psychological distress. This scale shows strong internal consistency and reliability for each subscale [48,72,73]. The DASS-21 has been validated in Australian adolescents [72], and in cancer patients [74].

AYA participants will also complete the *KIDCOPE (Older version)*[75], in order to assess their positive and negative coping approaches.

#### Family functioning measures

Both AYA participants and support-person participants will complete the general functioning, family communication, and problem-solving subscales of the *McMaster Family Assessment Device*[76].

#### Intervention engagement and impact

To assess AYAs’ mood in a more dynamic manner across the intervention, AYAs will complete the emotion thermometers tool [77] each week, 24-hours prior to participating in the weekly intervention session (RL or PSG). At the same time, AYA participants will complete the 6-point *Homework Compliance Scale*[78], to assess compliance with the home practice exercises. Support persons will also complete the weekly emotion thermometers tool, included with their weekly psycho-educational email.

#### Satisfaction with intervention

The brief *Youth Satisfaction Questionnaire* (YSQ) [79,80], will be completed by both AYA and support-person participants to assess their satisfaction with care and overall experience. At T2 and T3 assessments, this will be followed by 10 ratings of specific intervention elements to determine their acceptability (RL and PSG). Open-ended questions will also be used to elicit their experiences in the program and suggestions for improvement.

#### Feasibility outcome measures

The feasibility of Recapture Life-AYA will be determined through a number of indices, including: (i) the proportion of participants who would have been able to participate in Recapture Life-AYA without the provision of any additional technology; (ii) the time taken to complete and return questionnaires at each time point; (iii) the study response rate; (iv) the attrition rates of the study as a whole and to the Recapture Life-AYA arm specifically; (v) completion rates for the two intervention arms; (vi) the proportion of AYAs who nominate a support person to participate. The feasibility of implementing the Recapture Life-AYA study procedures will be assessed by examining the flow through the study (from opt-in to T3 assessment completion), using medians and ranges at each point.

### Data management and analysis

All measures (except the telephone interview) will be administered online, through Lime Survey^TM^ at all timepoints. This secure tool enables participants’ data to be downloaded to files amenable to statistical analysis, such as the Statistical Package for the Social Sciences (SPSS, by IBM).

#### Statistical analyses

This trial will employ ‘Intention to treat’ and ‘as treated’ analyses. Analyses will be based on mixed random-intercept models which will assess differences between the groups in terms of change in QoL from T1 to T2, and from T1 to T3. Random intercept models, which utilise maximum-likelihood estimation, provide more efficient estimates of effects with unbalanced data than the traditional repeated measures approach [81]. Analyses will follow recent commentaries on the best way to manage missing data by employing multiple imputation techniques [82]. Multiple regression analyses will also be conducted using T1 data to identify demographic and other factors that contribute to treatment outcome. Multiple comparisons will be used to test a priori and post-hoc hypotheses, appropriately adjusted to maintain the nominated Type I error rate.

## Discussion

This paper outlines the protocol for a multicenter RCT of a novel CBT-based, online intervention for adolescent and young adult cancer survivors. AYAs have been acknowledged as the ‘lost tribe’ or ‘forgotten generation’ in supportive cancer care [83,84]. Given their documented psychological vulnerabilities, Recapture Life-AYA addresses a critical gap for AYAs living with cancer. The innovative online technology used to deliver Recapture Life-AYA is an important advance, as it enables the provision of evidence-based psychological support to AYAs dispersed across both pediatric and adult hospitals, in metropolitan, rural and remote regions. This is crucial to overcoming both AYAs’ lack of ‘critical mass’ as a population [19], as well as the ‘tyranny of distance’ that impedes adequate post-cancer support more generally in countries with a low population density, such as Australia [85].

A further strength of this study is the inclusion of both an active and a waitlist control group. The waitlist group controls for the possibilities that AYA distress may dissipate in the first weeks after treatment completion and/or that clinical services may change or improve over the recruitment period. However, the additional use of a non-specific treatment arm (the PSG) is now considered gold standard, as this arm holds constant the attention received by participants, amount of treatment contact and human interaction variables (such as clinician warmth and interaction between participants), and controls for participants’ expectations of receiving some form of treatment [86]. This trial therefore allows an assessment of whether an intensive, structured, skills-based intervention such as Recapture Life-AYA confers any benefit in long-term psychological functioning relative to peer support alone. This question has important clinical, as well as economic considerations, as the two strategies have different implications for resources, training and time commitments required.

In addition, the strict treatment fidelity assessments involved in this trial will enable an examination the relative benefits of a structured, CBT-based intervention when compared with a non-directive, peer support group model. It is possible and likely that although the psychologist facilitator does not teach the same structured, CBT-based coping skills in the PSG, peer-discussion may nevertheless facilitate spontaneous discussion around adaptive coping skills, maladaptive/unhelpful thinking styles, or stress reduction strategies for example. The treatment fidelity assessments, including the coding of a random sample of 15% of sessions for cognitive content, will ensure an examination of the extent to which such skills-based discussion is facilitated, or directed, by the Recapture Life-AYA facilitator, and the proportion of session time spent on discussing adaptive coping strategies. Consequently, although there may be a reasonable degree of content overlap between the PSG and the Recapture Life-AYA groups, the rigorous treatment fidelity processes in this trial will mean that it is still possible to distinguish between the two arms in terms of mechanisms of change. This is critical in order to make recommendations for future intervention design.

The innovative videoconferencing delivery method used in Recapture life involves inherent challenges. There is growing evidence supporting the promise of this method in treating a number of disorders (e.g., Obsessive-Compulsive Disorder) [87]. Evidence suggests that several core aspects of CBT such as cognitive challenging, role-playing and modeling, setting up behavioural experiments and homework assignments, will translate well over videoconferencing methods [88]. Further, videoconferencing methods do not appear to diminish therapist competence, adherence, or patient perceptions of rapport or empathy conveyed by the therapist [89]. However, as most ‘online therapies’ are self-guided by the user with either telephone/email-based clinician support [90], few manualised treatment programs or best-practice guidelines exist to support the therapist in aspects relating to therapy process and online interaction. This may mean that therapist practice effects occur across the study period, as the therapist gains competency in anticipating, and managing, challenges in the videoconferencing environment.

One challenge inherent in the design of this study will be to quantify the effect of support person (e.g., parent/caregiver) participation on AYA psychological outcomes. Parents/caregivers may have an important role in assisting AYAs to negotiate the disruption cancer causes in their normal trajectory toward independence [91]. Parent inclusion in skills-based programs also appears to increase their likelihood of improving AYA psychological outcomes [29]. However, in this trial the decision was made to make parent/support person participation optional, to facilitate and support the autonomy and independence of the AYA participants. This design may result in greater statistical complexity when attempting to delineate the impact of parental/support person involvement in AYA interventions.

Despite its strengths, the study design also has methodological vulnerabilities. The three-armed design of this trial, while a strength on the one hand, is also likely to increase the time it takes to recruit sufficient participant numbers. In addition, our decision to divide AYA participants into two defined age groups (15–18 and 19–25 years) may increase the time between recruitment and actual group participation.

The long-term, 12-month follow-up is another aspect of the study design that, although methodologically important, may add complexities to final data analyses. Participants may differ markedly in terms of the psychosocial support services they receive in this time, and the number of other AYA cancer survivors they come into contact with. Individual differences in terms of additional support services and peer support is a factor that is likely to be important throughout the trial as a whole. All of these factors will require careful monitoring and documentation, and will need to be taken into account in data analyses and interpretation.

### Conclusions

Adolescent and young adult cancer survivors are a vulnerable population. Poor adaptation in this group threatens adaptive functioning in adulthood, for years after surviving cancer. Recapture Life-AYA is a preventative program with the potential to avoid many years of mental health problems by equipping AYAs with coping skills to successfully manage the challenges of survivorship. This study trials a new model of healthcare delivery that can extend the reach of support to isolated populations world-wide. If this study demonstrates significant improvements in quality of life, Recapture Life-AYA will be made available for clinical use with AYA cancer survivors across Australia.

## Abbreviations

ARM: Adolescent Resilience Model; AYA: Adolescent and young adult; CBT: Cognitive behavioural therapy; DASS21: Depression anxiety and stress scale-short form; PAIS: Psychosocial adjustment to illness scale; PSG: Peer support group; QoL: Quality of life; RCT: Randomised controlled trial; ReCaPTure LiFe: Resilience and Coping Skills for young People to Live well Following cancer; RL: ReCaPTure LiFe; SPSS: Statistical package for the social sciences; USD: Ursula Sansom-Daly psychologist; YSQ: Youth satisfaction questionnaire.

## Competing interests

The authors declare that they have no competing interests.

## Authors’ contributions

USD, CW, RB, PB and RC developed the study concept, aims and initiated the project. **SS,** PP, AA, and KT assisted in the further in depth development of the protocol. USD was responsible for the drafting of the manuscript. USD, CW, SS, AA, KT and RC will implement the protocol and oversee collection of the data. All authors have read and approved the final manuscript.

## Pre-publication history

The pre-publication history for this paper can be accessed here:

http://www.biomedcentral.com/1471-2407/12/339/prepub

## Supplementary Material

Additional file 1 **Table S1.** Recapture Life intervention components according to the Adolescent Resilience in Illness Model [adapted from 55]. Click here for file

Additional file 2 **Table S2.** Recapture Life-AYA intervention session content. Click here for file

Additional file 3 **Table S3.** Safety monitoring procedures. Click here for file

Additional file 4 **Table S4.** Description of assessment measures. Click here for file
