# Supplementary material for: Online group-based cognitive-behavioural therapy for adolescents and young adults after cancer treatment: A multicenter randomised controlled trial of Recapture Life-AYA
Source: BMC Cancer. 2012 Aug 3;12:339. doi: 10.1186/1471-2407-12-339 (PMC3503656; doi:10.1186/1471-2407-12-339)
Supplement: Additional file 1 — Table S1. Recapture Life intervention components according to the Adolescent Resilience in Illness Model [adapted from 55]. [file 1471-2407-12-339-S1.docx]

**TABLES AND FIGURES**

*Additional Table 1: Recapture Life intervention components according to the Adolescent Resilience in Illness Model [adapted from 55]*

| **Factor** | **Concept** | **Modifiable components** | **Relevant Recapture Life-AYA program strategies** |
| --- | --- | --- | --- |
| **Illness-related risk** | Uncertainty in illness | Perceived illness ambiguity and complexity | - Peer discussion to normalise range of typical AYA experiences - Behavioural activation to improve mood, fatigue, and activity levels - Cognitive strategies to change how AYAs engage with distressing illness-related thoughts |
|  | Disease-related distress | Symptom distress (pain, anxiety, fatigue, mood) |  |
| **Family protective** | Family atmosphere | Adaptability and cohesion | - Peer discussion and normalization around common challenges for families and parent-adolescent relationships - Cognitive challenging around unhelpful thoughts (AYA-parent interactions) - Assertive, effective communication skills strategies - Cognitive and behavioural strategies to proactively elicit support whilst negotiating desired boundaries/autonomy |
|  |  | Parent-adolescent communication |  |
|  |  | Perceived social support from family |  |
|  | Family support and resources | Family network | - Supportive discussion and problem-solving strategies to stimulate support seeking from family and network - Information and resources relating to additional available supports for AYA and their family |
| **Social protective** | Social integration | Perceived social support from friends | - Peer discussion and normalizing of common AYA experience of returning to social groups after cancer - Cognitive challenging and reappraisal skills (social support/friends) - Behavioural strategies to increase social support |
|  |  | Influence from other AYAs with cancer | - Peer discussion throughout program |
|  |  | Attitudes of illness disclosure | - Discussion and normalizing common experiences - Cognitive strategies to manage difficult thoughts/ emotions around this topic as well as behavioural strategies to manage illness disclosure situations. |
|  | Health care resources | Perceived support from providers | - Discussion and communication skills training around seeking advice and support from health care providers |
|  |  | Adolescent support program participation | - Recapture Life program participation (overall) - Linkage to other available information and resources |
| **Individual risk and**  **protective** | Defensive coping | Evasive, emotive and fatalistic coping | - Cognitive challenging of unhelpful thoughts - Acceptance- (rather than avoidance-based) strategies to manage confronting cancer-related thoughts - Behavioural strategies to decrease avoidance of cancer-related reminders - Behavioural strategies to increase proactive engagement with valued activities in AYAs’ lives |
|  | Courageous coping | Confrontive, optimistic, and supportant coping |  |
|  | Derived meaning | Hope  Post-traumatic/personal growth | - Cognitive strategies (e.g., challenging, reappraisal) to promote adaptive integration of cancer experience - Interaction with similar peers to stimulate sense of hope and new ways of coping |
| **Adaptive outcome** | Resilience | Confidence/mastery | - Cognitive strategies to challenge unhelpful thoughts relating to ability to navigate life after cancer - Cognitive and behavioural strategies to highlight existing and new strengths after cancer - Discussion with similar peers to share adaptive ways of coping and overcoming survivorship challenges |
|  |  | Self-transcendence  (sense of self following, and in context of, illness) | - Discussion around, and cognitive reappraisal strategies to facilitate, new ways of viewing cancer experience in context of life to date and imagined future - Behavioural strategies to stimulate re-engagement with valued activities to facilitate sense of a continuous self |
|  |  | Self-esteem | - Contact with other AYAs with cancer to boost sense of normalcy and social support - Cognitive and behavioural skills to reconnect with old friends and previous routines/activities after cancer - Cognitive and behavioural strategies to explore new life aspects to promote sense of wellbeing in survivorship |
|  | Quality of life | Sense of well-being | - Cognitive and behavioural strategies to integrate aspects of adapting to life after cancer (as above) |
