# Supplementary material for: Online group-based cognitive-behavioural therapy for adolescents and young adults after cancer treatment: A multicenter randomised controlled trial of Recapture Life-AYA
Source: BMC Cancer. 2012 Aug 3;12:339. doi: 10.1186/1471-2407-12-339 (PMC3503656; doi:10.1186/1471-2407-12-339)
Supplement: Additional file 2 — Table S2. Recapture Life-AYA intervention session content. [file 1471-2407-12-339-S2.docx]

**TABLES AND FIGURES**

*Additional Table 2: Recapture Life-AYA intervention session content*

| **Module** | **Psychological objectives/skills** | **Cancer relevant content** | **Support person update focus** |
| --- | --- | --- | --- |
| PSYCHO-EDUCATION | - Program engagement - Understanding value of learning positive coping skills - Normalising concerns/fears | Common experiences during & after cancer. What ‘survivor’ and ‘cure’ means. What ‘getting back to normal’ involves. How simple coping skills can help. | Common experiences and challenges during & after cancer for AYAs and their families/partners. Range of normal emotions & reactions at this time. |
| HEALTHY LIVING | - Understanding the importance of building structure and positive events back into life - Brainstorming ways to do this | Getting back in the driver’s seat after cancer: education, friends, healthy lifestyle as stress management. Addressing challenges (e.g. fatigue). | Overview of the importance of healthy lifestyles and the ‘interrupting’ effect that cancer can have on this. The link between mood, energy and activity levels. |
| APPRAISAL | - ‘ABC model’ of how when faced with an activating event, beliefs/thoughts impact consequences (feelings/actions) | Thoughts and feelings after cancer: changes to routine, body, friends, family. Impact on self-esteem. Impact of thoughts on how we feel and behave now. | Simple overview of ‘ABC’ model and the relevance of thinking about ‘how we think about cancer’ and its impact on our lives. Brief overview of thought challenging. |
| ACCEPTANCE | - Learning to evaluate the usefulness of different types of thoughts (e.g., worry) - Learning what to thought challenge and what to accept - Strategies to reduce distress and enhance acceptance | Fear of cancer recurrence and the long-term effects of cancer, existential thoughts. Strategies that they have tried to deal with these challenging thoughts – what has worked and what hasn’t. | What fear of cancer recurrence is and how it can manifest. Why thoughts like this might be daunting and distressing for both AYAs and support persons. Simple overview of the different strategies taught to deal with these kinds of thoughts. |
| SOCIAL SUPPORT | - Rationale behind improving communication skills and social support, regardless of current mood - Skills practise | Reconnecting with friends after cancer; how to talk about cancer. Simple skills to navigate difficult social situations post-cancer. Workshopping cancer-related problems. | Overview of some of the effects cancer can have on young people’s social lives, and the effects this has on their mood. Overview of skills including assertive communication tips. |
| GOAL-SETTING & REFRAMING | - Rationale for setting future goals - Apply appraisal & problem solving to goals - Preparing for survivorship | Discussion of pre-cancer/new goals. What being a ‘survivor’ means for goals. Using skills to reconcile goals that may now be more difficult (e.g., financial, employment challenges). Accessing advocacy, information, support. | Disruption that cancer can cause for goals for all family members. How skills taught in the AYAs’ previous sessions can be used to help cope with these changed goals. |
