# Supplementary material for: Online group-based cognitive-behavioural therapy for adolescents and young adults after cancer treatment: A multicenter randomised controlled trial of Recapture Life-AYA
Source: BMC Cancer. 2012 Aug 3;12:339. doi: 10.1186/1471-2407-12-339 (PMC3503656; doi:10.1186/1471-2407-12-339)
Supplement: Additional file 3 — Table S3. Safety monitoring procedures. [file 1471-2407-12-339-S3.docx]

*Table 3: Safety monitoring procedures*

| **Stage of trial** | **Measure of risk*** | **How determined** | **Follow-up procedures** |
| --- | --- | --- | --- |
| Recruitment | Intake interview: validated *Adolescent Suicidal Assessment Protocol* items  (suicidal risk, psychosis) | - Endorse serious, current suicidal or homicidal ideation - Report current delusions/hallucinations | - Immediate cessation of intake, discussion about participant’s emotional state with study psychologist - Discussion of relevant supports (e.g., nominated GP) and plan made for AYA to contact these - Provision of emergency numbers, urged to contact these in an emergency - Referral back to nominated primary health care professional (HCP) |
| All assessments  (T1, T2, T3) | DASS21 depression subscale | - Score >28 (extremely severe) on depression subscale; *and/or* - Report that *“I felt that life was meaningless”* Very Much or Most of the Time (DASS21 item 13) | - Study exclusion - Participant telephoned to discuss their current emotional state and to conduct a suicide risk assessment - Meeting of study investigators to discuss and document a management plan |
| During intervention | Emotion thermometers tool | - Rate an emotion ≥7 out of 10; *and/or* - Record a change in one of these emotions more than three points | - Participant telephoned to discuss their current emotional state and to conduct a suicide risk assessment - Meeting of study investigators to discuss and document a management plan |
| General precautions throughout trial | Study communications | - N/A – risk determined only if AYA participant reports it to study coordinators | - Emergency numbers and information about what to do in the case of acute distress in study email footers - Same information also in Recapture Life-AYA workbook |

*Risk defined as significant risk of harm to themselves or another person
