# Supplementary material for: Online group-based cognitive-behavioural therapy for adolescents and young adults after cancer treatment: A multicenter randomised controlled trial of Recapture Life-AYA
Source: BMC Cancer. 2012 Aug 3;12:339. doi: 10.1186/1471-2407-12-339 (PMC3503656; doi:10.1186/1471-2407-12-339)
Supplement: Additional file 4 — Table S4. Description of assessment measures. [file 1471-2407-12-339-S4.docx]

*Table 4: Description of assessment measures*

| **Domain** | **Construct** | **Measure name** | **No. of items** | **Validated?** | **Cancer-specific?** | **Description** |
| --- | --- | --- | --- | --- | --- | --- |
| Demographics | Patient information | Demographic data | 7 | No | Yes | Assesses: participant age, sex, education, employment status, family structure, cancer diagnosis, treatment regimen, and time since treatment. |
| Psychosocial Functioning | Health-related quality of life | Impact of Cancer Scale (IOCS)^^^ | **54** | Yes | Yes | Assesses: physical/psychosocial health, emotional, social and work functioning, and cancer-specific issues such as health literacy, fear of recurrence, cognitive problems, perceived appearance and communication issues. |
|  | Adjustment to chronic illness | Psychosocial Adjustment to Illness Scale-Interview form (PAIS) | 46 | Yes | Yes | Clinical interview assessing adjustment over 7 domains: Health care orientation, Vocational environment, Domestic environment, Sexual relationships, Extended family relationships, Social environment, Psychological distress. |
|  | Mood and emotional state | Emotion thermometers tool | 5 | Yes | No | Assesses: four predictor domains (distress, anxiety, depression, anger) and one outcome domain (need for help). Each domain is rated on a 0 to 10 point Likert scale in a visual thermometer, |
|  | Depression, anxiety, stress symptoms | Depression, Anxiety, Stress Scales-short form (DASS-21) | 21 | Yes | No | Assesses: distress across 3 subscales (depression, anxiety and stress), of 7 items each. Respondents use a 4 point scale to rate how often they have experienced each symptom in the past week (“Not at all” to “Most of the time”). |
|  | Impact of cancer event on sense of self | Centrality of Event Scale-Short Form | 7 | Yes | No | Assesses: how central an event is to a person’s identity and life story. Can be used for any stressful or traumatic event specified by the participant (e.g., cancer diagnosis). |
|  | Cancer-related self-identity | Perception as “cancer survivor” item | 2 | No | Yes | Assesses: what cancer-related label or description the young person most identifies with (six options), and the extent to which they identify themselves as a patient or a survivor (10 point Likert scale). |
|  | Coping strategies | KIDCOPE-Older Version | 10 | Yes | No | Assesses: positive and negative adolescent coping approaches. Respondents name a recent cancer-related problem and rate 10 coping strategies for frequency of use and helpfulness. |
| Family Functioning | Family-level functioning, communication and problem-solving | McMaster Family Assessment Device* | 23  (of 53 total) | Yes | No | Assesses: 7 aspects of family functioning: problem-solving, communication, roles, affective responsiveness, affective involvement, behaviour control, and general functioning. A 4-point Likert response format is used. The subscales of family communication (6 items), problem-solving (5 items), and general functioning (12 items) will be used in this study. |
| Intervention engagement | Homework completion | Homework Compliance Scale | 6 | Yes? | No | Assesses: extent of homework completion in previous week. |
|  | Satisfaction with clinical services | Youth Satisfaction Questionnaire | 5 | Yes | No | Assesses: satisfaction with care and overall experience following cancer treatment completion, including help needed and received. Only the general satisfaction section will be administered for this study. |
|  | Satisfaction with intervention received | Intervention satisfaction items | 10 | No | Yes | Assesses: participants’ satisfaction with specific aspects of the intervention including content, skills taught/learnt, workbook, peer discussion, and online delivery method. |

^ NB: for IOCS questions pertaining to sexuality in this questionnaire, an additional option of “I would prefer not to answer this question” has been added, to accommodate the younger participants in the study.
